# Compositional Design of Dielectric, Ferroelectric and Piezoelectric Properties of (K, Na)NbO_3_ and (Ba, Na)(Ti, Nb)O_3_ Based Ceramics Prepared by Different Sintering Routes

**DOI:** 10.3390/ma9030179

**Published:** 2016-03-08

**Authors:** José A. Eiras, Rosimeire B. Z. Gerbasi, Jaciele M. Rosso, Daniel M. Silva, Luiz F. Cótica, Ivair A. Santos, Camila A. Souza, Manuel H. Lente

**Affiliations:** 1 Physics Department, Federal University of São Carlos, São Carlos SP 13565-905, Brazil; 2 Physics Department, Maringá State University, Av. Colombo, 5790, Maringá PR 87020-900, Brazil; rosizampiere@hotmail.com (R.B.Z.G.); jaci_rosso@hotmail.com (J.M.R.); danieldmats@gmail.com (D.M.S.); lfcotica@dfi.uem.br (L.F.C.); iasantos@dfi.uem.br (I.A.S.); 3 Science and Technology Institute, Federal University of Sao Paulo, 330 Talim street—Vila Nair, São Jose dos Campos SP 12231-280, Brazil; camialvesdesouza@gmail.com (C.A.S.); mlente@unifesp.br (M.H.L.)

**Keywords:** lead-free piezoelectrics, ferroelectrics, piezoelectrics, spark plasma sintering

## Abstract

Lead free piezoelectric materials are being intensively investigated in order to substitute lead based ones, commonly used in many different applications. Among the most promising lead-free materials are those with modified NaNbO_3_, such as (K, Na)NbO_3_ (KNN) and (Ba, Na)(Ti, Nb)O_3_ (BTNN) families. From a ceramic processing point of view, high density single phase KNN and BTNN ceramics are very difficult to sinter due to the volatility of the alkaline elements, the narrow sintering temperature range and the anomalous grain growth. In this work, Spark Plasma Sintering (SPS) and high-energy ball milling (HEBM), following heat treatments (calcining and sintering), in oxidative (O_2_) atmosphere have been used to prepare single phase highly densified KNN (“pure” and Cu^2+^ or Li^1+^ doped), with theoretical densities ρ_th_ > 97% and BTNN ceramics (ρ_th_ ~ 90%), respectively. Using BTTN ceramics with a *P*4*mm* perovskite-like structure, we showed that by increasing the NaNbO_3_ content, the ferroelectric properties change from having a relaxor effect to an almost “normal” ferroelectric character, while the tetragonality and grain size increase and the shear piezoelectric coefficients (*k_15_*, *g_15_* and *d_15_*) improve. For KNN ceramics, the results reveal that the values for remanent polarization as well as for most of the coercive field are quite similar among all compositions. These facts evidenced that Cu^2+^ may be incorporated into the A and/or B sites of the perovskite structure, having both hardening and softening effects.

## 1. Introduction

Since the discovery of ferroelectric ceramics in the 1940s, their piezoelectric properties have been used in many electronic devices such as sensors, actuators and ultrasound transducers [1]. Among them, lead based ferroelectric systems such as, for example, Pb(Zr_1−*x*_Ti*_x_*)O_3_ (PZT), Pb(Mg_1/3_Nb_2/3_)O_3_-PbTiO_3_ (PMN-PT), Pb(Zn_1/3_Nb_2/3_)O_3_-PbTiO_3_ (PZN-PT) and some related ones, present the highest performances [2,3]. Notably, these materials have been successfully used in many technologically advanced applications that have helped in raising the population’s quality of life, leading to important advances in several areas, such as health and energy harvesting [4]. However, from an environmental point of view, the replacement of lead-containing materials/devices is imperative, even if there is some loss of performance in existing devices, because during the fabrication process (calcination, sintering) of these compounds, a release of poisonous lead can occur causing environmental and health contamination. In the last decade, the international scientific community has been investigating several lead-free piezoceramic compositions to replace Pb [5,6,7], working to gain equivalent or even superior properties to those of Pb-based materials [8].

Regarding the crystal structure, surely lead-free ferroelectric perovskites present much higher piezoelectric properties than those observed in lead-free products with a bismuth layer or tungsten bronze structures [9]. Nevertheless, there are no single lead-free perovskite compositions with piezoelectric performance comparable to those observed in PZT or PMN-PT ceramics. Thus, possible candidates to substitute the Pb-piezoceramics are those Pb-free compositions based on binary, ternary or even quaternary systems [10].

Among the innumerous alternatives for substituting Pb-containing perovskite ferroelectric materials for lead-free ones, the most promising candidates are those based on solid solutions between the antiferroelectric NaNbO_3_ compound and other ferroelectric compounds, such as KNbO_3_ and BaTiO_3_, for example [11,12], forming KNbO_3_-NaNbO_3_ [(K_x_Na_1−x_)NbO_3_-KNN] [13,14,15,16] and BaTiO_3_-NaNbO_3_ [(Ba*_x_*Na_1−*x*_)(Ti*_x_*Nb_1−*x*_)O_3_-BTNN] [14,15,16,17,18,19,20,21] solid solutions. These two systems form a very interesting and complementary ensemble of piezoceramics that cover most of the technological demands of actuator and ultrasonic transducer applications. For instance, by carefully controlling the doping amount of NaNbO_3_ going into the BaTiO_3_, it is possible to produce piezoceramics with piezoelectric coefficients higher than those found in PZT ceramics, although with a lower Curie temperature. In contrast, modifying KNbO_3_ with NaNbO_3_ produces piezoceramics with lower piezoelectric coefficients but with a higher Curie temperature [17]. Therefore, exhausting efforts are being employed to optimize the physical properties of KNN and BTNN ceramics by suitable chemical modifications and innovative processing routes.

Similar to PZT ceramics, KNN and BTNN systems can form complete solid solutions where the effects and impacts on physical properties due to iso- and/or heterovalent substitutions in both A and B perovskite sites can be exploited. Moreover, KNN presents a rich and interesting phase diagram with a morphotropic phase boundary (MPB), namely at around 50:50 mol [18]. When the Na/K ratio is around 1, the piezoelectric and dielectric properties of KNN present better properties compared to other Na/K contents [6,19,20]. However, high density KNN ceramics are difficult to produce due to the volatility of K/Na elements, narrow sintering temperature range, anomalous grain growth and segregation of alkaline elements [21,22,23,24]. In order to overcome these problems, some processing methods such as hot pressing and spark plasma sintering have been used to improve the microstructural and structural properties [25,26,27,28,29]. Nevertheless, even by using advanced processing methods, for KNN piezoceramics, very often no reproducibility in the microstructural and structural phases can be reached, as pointed out by some authors [30]. One of the reasons for this fact is the influence of the Nb_2_O_5_ phase on the structural and microstructural properties of KNN [28,31]. Moreover, the dielectric and piezoelectric properties of KNN may be improved by adding dopants such as Li^+^ (A-site) or Cu^2+^ (B-site). It is believed that these elements induce, respectively, soft and hard piezoelectric behaviors. In particular, it has been proposed that the Cu^2+^ may occupy both the A and B sites in the perovskite lattice, leading to opposite behaviors in their electrical properties [32,33].

On the other hand, (Ba*_x_*Na_1−*x*_)(Ti*_x_*Nb_1−*x*_)O_3_-BTNN compounds can present conventional or relaxor ferroelectric behaviors depending on the x amount in the solid solution [34,35,36]. Regarding the crystalline structure, BTNN compounds present a perovskite tetragonal symmetry for *x* < 0.30, a pseudo cubic symmetry for intermediate *x* values (0.30 ≤ *x* ≤ 0.70) and, with x increasing (0.70 ≤ *x* ≤ 0.88), the tetragonal symmetry emerges once again [14,37,38]. Finally, for x in the range of 0.90–0.96, an additional distortion occurs and the symmetry changes from a tetragonal to an orthorhombic one [14,39,40]. It is worth noting that the temperature of maximum dielectric constant (*T*_m_) decreases until *x* ~ 0.30. For higher *x* values, *T*_m_ increases with *x* increasing. Moreover, an elevated dielectric constant is observed for the majority of the compounds, which is highest (ε′_max_ = 9500) for *x* = 0.90 [14,15,16,17,18,19,20,21,22,23,24,25,26,27,28,29,30,31,32,33,34,35,36,37,38,39,40,41]. Considering all these aspects, *i.e.*, the existence of high dielectric constants, polar space groups (tetragonal and orthorhombic phases), relaxor behavior and high remanent polarizations, the potential for applying BTNN solid solutions in high capacitive, piezoelectric and ferroelectric devices naturally emerges.

From a ceramic processing point of view, high-density BTNN ceramics are also very difficult to sinter [22,23,24]. Rather, the BTNN system relies on both very long calcining times (up to 15 h) and elevated sintering temperatures (reaching 1450 °C) [31,32,33,34,35,36,37]. This enhances the volatilization of the precursor oxides, making densification difficult and increasing the time needed for ceramic processing. In this context, different processing strategies in the preparation processes are needed to obtain high-density single phase BTNN ceramics. For instance, the preparation of BTNN powders by mechanochemical activation in association to thermal treatments during the calcining and sintering steps have been revealed to be very attractive to avoid these limitations [42,43].

In this work, Spark Plasma Sintering (SPS) and high-energy ball milling (HEBM) followed by thermal treatments (calcining and sintering) in oxidative (O_2_) atmosphere have been used to prepare KNN, pure and Cu or Li doped, and BTNN ceramics, respectively. These procedures were successfully applied to produce single-phase high-densified ceramic bodies.

## 2. Experimental Section

Single-phase (1−*x*)BaTiO_3_-(*x*)NaNbO_3_ (BTNN) powders, with x ranging from 0.10 to 0.90, were synthesized from dry analytical reagent grade powders (Aldrich) of BaCO_3_, TiO_2_, Na_2_CO_3_ and Nb_2_O_5_. Polycrystalline BTNN lead-free ceramics were processed by dry high-energy ball milling (HEBM), in a Retsch PM100 planetary ball mill, followed by thermal treatments (calcining and sintering) in oxidative (O_2_) atmosphere. The milling parameters were as follows: ball-to-powder mass ratio was 12:1, the rotation speed of the supporting disc and vial was 32 rad·s^−1^, and the milling time of 3.0 h was fixed, as previously reported [44]. After milling, the powder samples were calcined in a tubular furnace at 1100 °C (0.40 ≤ *x* ≤ 0.90) and 1150 °C (0.10 ≤ *x* ≤ 0.30) for 1.0 h in O_2_ atmosphere. These powders were isostatic cold pressed (120 MPa) in disc shaped ceramic bodies and sintered in O_2_ atmosphere at temperatures ranging from 1200 °C to 1330 °C for 1.0 h.

Stoichiometric (Na_0.52_K_0.48_)NbO_3_ (KNN), (Na_0.52_K_0.48_)(Nb_0.985_Cu_0.015_)O_3_ (KNN + Cu) and (Na_0.495_K_0.455_Li_0.05_)NbO_3_ (KNN + Li) powders were prepared by a conventional mixed-oxide process by using K_2_CO_3_, Na_2_CO_3_ and Li_2_CO_3_ carbonates (Sigma-Aldrich, St. Louis, MO, USA, >99.0% purity), Nb_2_O_5_ (Sigma-Aldrich, 99.9%) and CuO (Sigma-Aldrich, 99.5% purity). It has been shown that for conventional sintering these amounts of Li^+^ and Cu^2+^ doping elements improve the sinterability of KNN piezoceramics [45]. Moreover, it is believed that Li^+^ and Cu^2+^ elements induce, respectively, soft and hard piezoelectric behavior of the doped piezoceramics.

The carbonate powders were dried in an oven for 3.0 h at 250 °C in order to eliminate adsorbed water. Then, the precursor powders were weighed according to the desired stoichiometry and the ball was mixed for 3.0 h in alcohol as liquid medium. The dried powders were calcined in air at 900 °C for 3.5 h. This temperature and time were found to be suitable to complete formation of the perovskite structure, as described in detail in a previous work [46]. Rather, it was shown recently that the formation of the perovskite structure starts at about 700 °C but, in regular synthesis processes, it ends at higher temperatures [47]. This behavior was verified in our previous studies [46].

A SPS furnace (Dr. Sinter 1020, SPS SYNTEX INC., Yokohama, Japan) was used for the sintering process for the KNN samples [48]. Briefly, the temperature was raised from room temperature to 1000 °C–1100 °C, depending on the composition, in Ar atmosphere and at a constant rate of 100 °C/min. The dwell time was 5 min for all experiments. An axial pressure of 50 MPa was applied at room temperature and kept constant during both the heating and the dwell times. After sintering, all the disc shaped ceramic bodies (9 mm in diameter and ~1 mm thick) were removed from the die and annealed in air at 850 °C for 3.5 h and polished.

XRD patterns were taken at room temperature on the BTNN powdered samples and KNN sintered ceramics (polished and annealed pellets). The bulk densities of the sintered samples were measured by the Archimedes method. In order to make electrical characterizations, silver paste (KNN) or gold (BTNN) electrodes were deposited onto the sintered sample faces. For piezoelectric characterization, the KNN samples were poled under an electric field of 35 kV/cm for 30 min at 90 °C in silicone oil. BTNN samples were poled with a poling field ranging from 32 kV/cm to 50 kV/cm for 0.5 h to 2.5 h, depending on the geometry of the samples and the electrodes’ configuration, as previously reported [49]. Piezoelectric characterizations were performed at room temperature by using the resonance–antiresonance method. The precision of the electrical measurements was within 5%–7%.

Dielectric measurements as a function of the temperature were performed using an Agilent Impedance meter. Ferroelectric hysteresis loops were characterized, at room temperature, in a Sawyer-Tower setup, applying a sinusoidal electric field, 3.0 Hz for the KNN and at 30 Hz for BTNN samples.

## 3. Results and Discussion

### 3.1. Structural and Microstructural Properties of BTNN and KNN Ceramics

**Figure 1:**
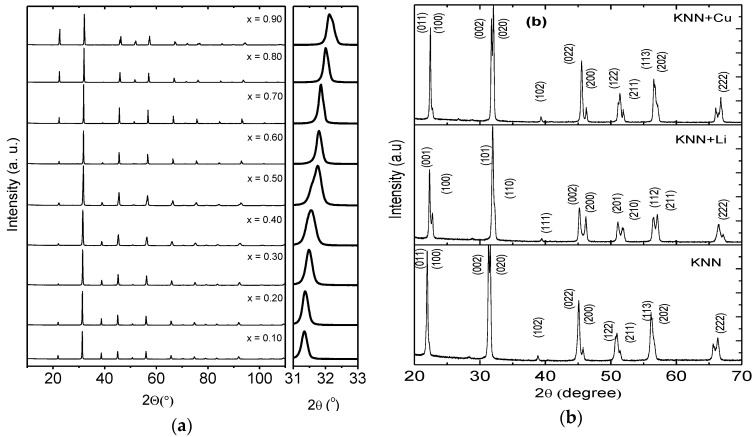
Room temperature X-ray diffraction patterns for (**a**) (1−*x*)BaTiO_3_-(*x*)NaNbO_3_powdered ceramics and (**b**) for “pure”, Li or Cu doped KNN ceramics.

Figure 1 shows the XRD patterns for all BTNN and KNN studied compositions. As can be seen, no spurious phases (in the detection limit of the used equipment) were detected in these samples. Contrary to what has been previously reported, where symmetries are composition dependent [14,15,16,17,18], all BTNN samples (Figure 1a) were crystallized in a single phase perovskite-like structure with a *P*4*mm* space group (determined following a le Bail refinement protocol). This results show the efficiency of the synthesis protocol (HEBM plus calcining and sintering in oxidative atmosphere) to produce structurally polar single-phase BTNN samples. As powder synthesis by HEBM introduces internal strains and other defects [14,15,16,17], it is believed that the related residual stresses tend to help in lowering the calcining temperatures and for retaining the tetragonal symmetry of the perovskite structure [50].

For the KNN ceramics (Figure 1b), the XRD patterns reveal that all ceramic bodies are in the perovskite phase without secondary phase within the detection limit of the used equipment.

For the KNN + Li samples, a more defined splitting of the XRD peaks in the region around 45.8° was observed, suggesting a change from a mixed orthorhombic-tetragonal phase to a more defined tetragonal one [51], which is consistent with the addition of Li to the KNN [52]. Some authors have reported the coexistence of phases in the (K*_x_*Na_1−*x*_)NbO_3_ ceramics [53,54]. On the other hand, the XRD pattern of KNN + Cu indicates a stabilization of the orthorhombic phase, promoted by the Cu^2+^ incorporation.

**Figure 2:**
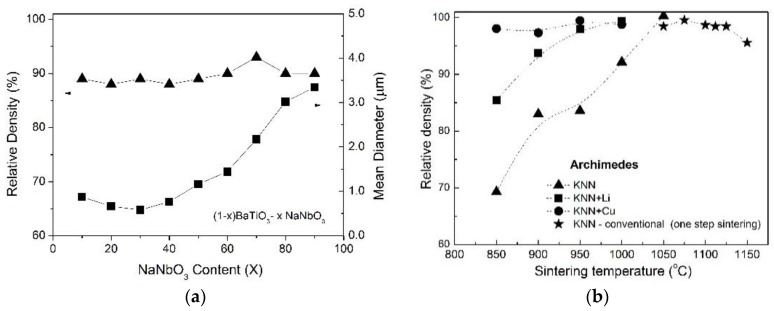
(**a**) Relative density (▲) and mean grain size diameter (■) for (1−*x*)BaTiO_3_-(*x*)NaNbO_3_ powdered ceramics as a function of the NaNbO_3_ content; (**b**) Relative density of “pure” and Li or Cu doped as a function of the sintering temperature for the KNN ceramics.

Figure 2a presents the evolution of the mean grain size diameter, determined by the average grain intercept method, and the relative density determined by the Archimedes method as a function of the NaNbO_3_ content (*x*) for the BTNN. Figure 2b depicts the sintering temperature dependence of the bulk density of the pure and doped KNN ceramic bodies.

As can be observed in Figure 2a, in the BTNN ceramics, the mean grain size decreases slightly until *x* ~ 0.30 and increases for *x* > 0.30, while the theoretical density remains nearly constant (ρ_th_ ~ 90%) for all compositions.

For the pure KNN and Li-doped ceramics, the bulk densities increased significantly with increases in the sintering temperature (Figure 2b), reaching around ρ_th_ ~ 97%. In contrast, for the Cu-doped KNN ceramics, the density is almost independent of the sintering temperature reaching ρ_th_ ~ 99.5%. For the optimized conditions (higher densities), the values found for the densities are similar to those found by other authors [25,55]. Moreover, in comparison to undoped KNN, it was verified that the Li or Cu addition reduced the sintering temperature. For the sake of comparison, results obtained through the conventional sintering route are also included in Figure 2b. It is important to stress that a direct comparison between the sintering temperatures, by conventional and SPS methods, should be avoided since, in the SPS system, the temperature is determined using a pyrometer taking measurements at the mold surface. It is difficult to determine the effective temperature reached in the samples using this procedure. However, the qualitative difference between the temperature dependence of the density by comparing both routes is clear. Moreover, it is possible to suggest that the KNN samples sintered at lower temperatures present predominantly open porosity, which gradually is closed with increasing sintering temperature.

**Figure 3:**
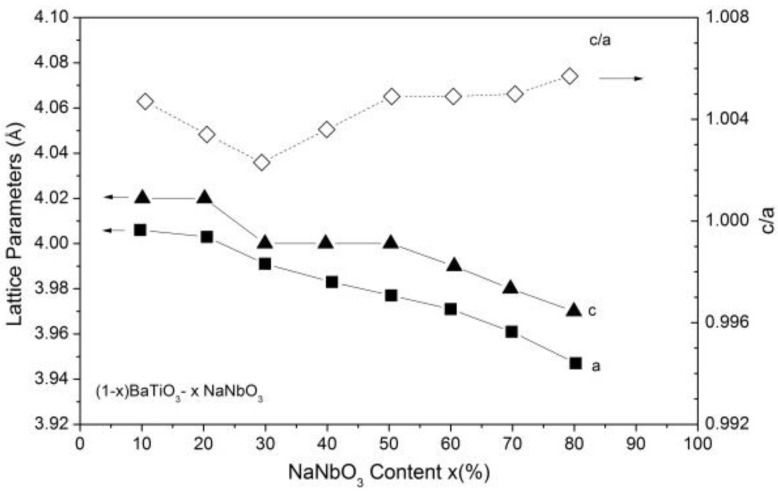
Lattice parameters a (■), c (▲) and tetragonality factors c/a (◊) for (1−*x*)BaTiO_3_-(*x*)NaNbO_3_ powdered ceramics as a function of *x*.

The chemical modification dependence of the lattice parameters (*a* and *c*) and tetragonality factors (*c/a*) of the (1−*x*)BaTiO_3_-(*x*)NaNbO_3_ samples are shown in Figure 3. As can be observed, both lattice parameters decreased with increasing x, while the tetragonality factor decreases slightly until *x* ~ 0.30 and increases for *x* > 0.30. This behavior is similar to the x dependence of the mean grain size observed in Figure 2. These results also reveal that the tetragonality increases as the grain size increases.

### 3.2. Dielectric, Piezoelectric and Ferroelectric Properties of BTNN

**Figure 4:**
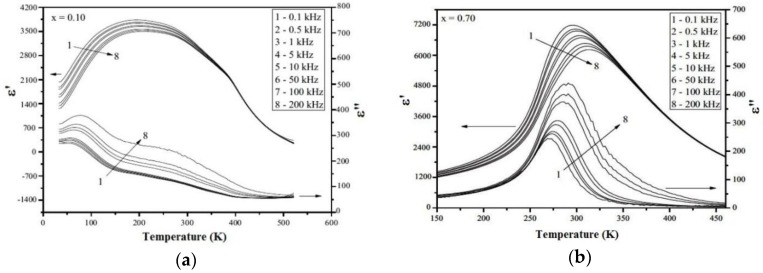
Real and imaginary parts of the dielectric constant as a function of the temperature, measured at different frequencies, for (**a**) 0.90BaTiO_3_-0.10NaNbO_3_ and (**b**) 0.30BaTiO_3_-0.70NaNbO_3_ samples.

Figure 4 shows the real (ε′) and imaginary (ε′′) parts of dielectric constant as a function of the temperature for the BTNN. For the sake of simplicity, only representative results obtained for 0.10BaTiO_3_-0.90NaNbO_3_ and 0.30BaTiO_3_-0.70NaNbO_3_ samples are shown. The real part of the relative dielectric curves reveals a broad peak typical of a relaxor-like behavior for all BTNN investigated samples.

**Figure 5:**
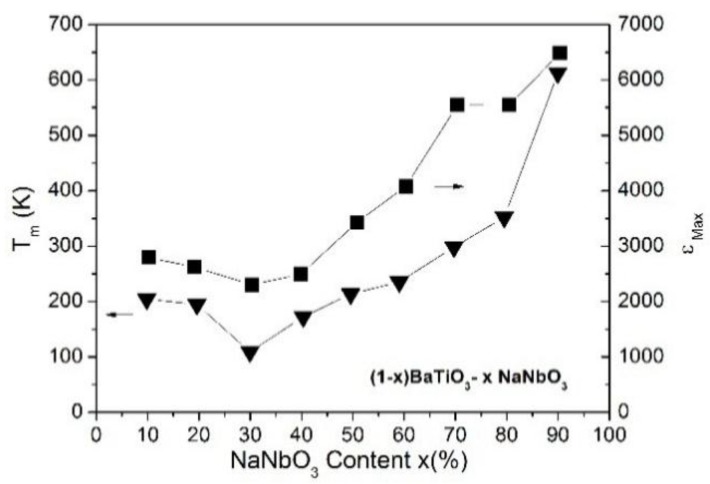
Maximum of the dielectric constant (peak) ε_Max_ (■) and the related temperature *T_m_* (▼) as a function of *x* for (1−*x*)BaTiO_3_-(*x*)NaNbO_3_ ceramic samples.

The chemical modification dependence of the maximum dielectric constant (ε′_max_) and its corresponding temperature (*T*_m_) for all investigated samples are shown in Figure 5. The observed values for *T*_m_ and ε′_max_ are in accordance with those reported previously for BTNN samples, processed by different milling and sintering protocols [31,32,33,34,35,36,37,38,44]. Similarly to that observed for the *x* dependence of the tetragonality factor (Figure 3), both parameters decrease for x ranging from 0.10 to 0.30 and increase for *x* > 0.30 up to ε′_max_ = 7500 and *T*_m_ = 648 K for the 0.10BaTiO_3_-0.90NaNbO_3_ sample. The ε′_max_ and *T*_m_ values and the evolution of the ferroelectric character (from relaxor to the almost normal ferroelectric), with the increase of *x*, can be correlated of the tetragonality factor of the studied samples.

**Figure 6:**
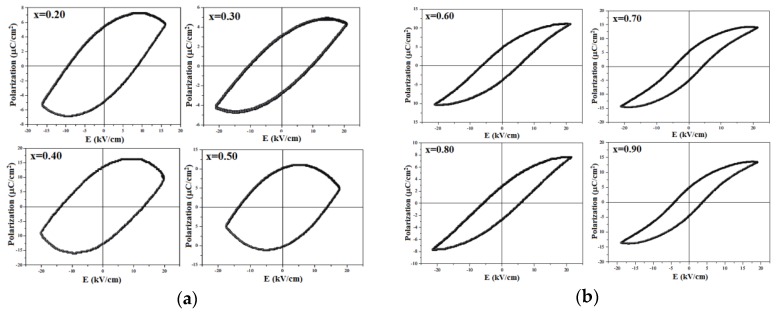
Ferroelectric hysteresis loops, determined at 30 Hz at room temperature, for (1−*x*)BaTiO_3_-(*x*)NaNbO_3_ ceramics, (**a**) 0.20 ≤ *x* ≤ 0.50 and (**b**) 0.60 ≤ *x* ≤ 0.90.

Ferroelectric hysteresis loops obtained for BTNN samples at room temperature and at 30 Hz are shown in Figure 6. As can be seen, for low NaNbO_3_ concentrations (up to *x* = 0.50), strong dissipative hysteresis loops are observed, preventing the correct determination of the ferroelectric parameters (Figure 6a). However, for *x* > 0.50, well-defined ferroelectric hysteresis loops are seen with *P*_r_ and *E*_c_ reaching 5 μC/cm^2^ and 5 kV/cm, respectively, for the *x* = 0.90 sample (Figure 6a). These results are in accordance with those reported in the literature [32,38,56], attesting the feasibility of the proposed HEBM protocol for producing BTNN ferroelectric samples. Furthermore, by comparing the results obtained for the *x* = 0.70 sample with those reported for Abdelkefi, *et al.* [51], a considerable increase of the ferroelectric response for the sample obtained by HEBM (*P*_r_ = 5 μC/cm^2^) is observed, as compared to that obtained by the conventional solid state method (*P*_r_ = 1.5 μC/cm^2^). These results may be attributed to the retention of the tetragonal symmetry (*P*4*mm* space group) in samples processed by HEBM.

Table 1 shows the relative dielectric permittivity and some electromechanical parameters, determined for the *x* = 0.70 to 0.90 BTNN samples using the resonance method. As a matter of comparison, reported values for PZT-EC-64 are also included. The majority of the BTNN piezoelectric coefficients are lower than those reported for the PZT-C-64 sample. This result can be attributed to the small room temperature tetragonality factor of these samples, which tend to reduce their ferroelectric and piezoelectric responses. However, it worth noting that some piezoelectric coefficients of the BTNN samples, such as the k_15_ electro-mechanical coupling factor, and the g_15_ and d_15_ reached values comparable with those for PZT-EC-64 (right column). This fact suggests that these samples can substitute PZT in practical piezoelectric applications, where shear modes are technologically exploited. Remarkable is the enhancement of the shear piezoelectric coefficient values (*d_15_*) obtained for the BTNN samples, compared to the longitudinal (*d_33_*) and extensional (*d_31_*) values. Further investigations are necessary to explain these results.

### 3.3. Dielectric, Piezoelectric and Ferroelectric Properties of KNN

**Figure 7:**
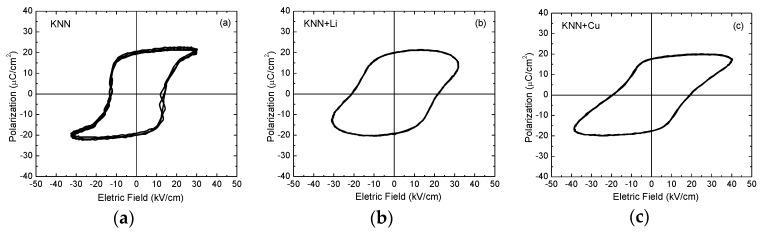
Ferroelectric hysteresis loops, measured at 3 Hz, for KNN ceramics (**a**) “pure”; (**b**) KNN + Li and (**c**) KNN + Cu doped ceramic samples.

Figure 7a–c shows, respectively, the ferroelectric hysteresis loops for the higher densified KNN, KNN + Li and KNN + Cu ceramic bodies. The results reveal that the values for both remanent polarization and mainly for the coercive field are quite similar among all compositions. The values found for the polarization and coercive field are similar to those found recently for soft KKN produced by SPS [26,27,57]. Considering the concept of softener and hardener elements in piezoceramics, a higher remanent polarization for the Li-doped KNN (“soft KNN”) and a ferroelectric constricted loop for Cu-doped KNN (“hard KNN”) would be expected in our data [58,59], as observed for soft and hard PZT ceramics [60]. Nevertheless, the shape of all ferroelectric hysteresis loops is quite similar. This fact suggests that some of the Cu^2+^ ions also entered the A-site, instead of the B-site, substituting partially the K^1+^ or Na^1+^ elements, thus being donor doping. In this case, it is believed that the Cu^2+^ ions may have a dual contribution to the ferroelectric and piezoelectric properties of KNN ceramics, with softening or hardening characteristics, depending on the occupation site. This behavior suggests that further improvements in the processing routes should be made to effectively control the site occupancy by Cu^2+^ ions.

Piezoelectric parameters for the “pure” KNN, KNN + Li and KNN + Cu ceramics are summarized in Table 2. The experimental results show that the *k*_p_ value of Li-doped KNN ceramics is slightly higher than those for pure or Cu-doped KNN ceramics. This result is expected since Li^+^ ions act as a softener element. However, the values obtained for mechanical quality factor (*Q*_m_) revealed intriguing results. In comparison to the “pure” and Li-doped KNN ceramics, it would be expected that the addition of Cu^2+^ ions should considerably increase the value of *Q*_m_, assuming that it is has a hardening characteristic. However, by comparing the values of *Q*_m_ for KNN + Li and KNN + Cu, it is observed that they are quite similar to each other. This experimental result suggests again, as mentioned earlier, that in our case, Cu^2+^ did not act truly as a hardener dopant in KNN ceramics.

Concerning the dual characteristic of Cu^2+^, in general, it is believed that the addition of CuO (Cu^2+^—73 pm) to the KNN ceramics may substitute the Nb^5+^ ions (0.64 pm) as an acceptor-type dopant, increasing the oxygen vacancy concentration, thus causing a hardening effect [54,61]. In this situation, several papers showed that CuO addition may significantly improve *Q*_m_ reaching sometimes values higher than 1500, which are much higher than that found in this work. As mentioned before, a possible explanation for such apparent discrepancy is to take into account that Cu^2+^ ions may not only enter the B-site in the KNN host lattice, but are also likely to enter the A-site or both, depending on the amount of Cu^2+^ ions and the sintering process. The ionic radius of Na^+^ (139 pm) or K^+^ (164 pm) allows such an assumption. By entering the A-site, each Cu^2+^ ion acts as a donor dopant rather than as a hardener due to its higher valence, compared to the substituted ion. Rather, such a hypothesis has been put forward by some authors [62,63,64,65]. Therefore, by comparing our ferroelectric and piezoelectric results, it seems that the Cu^2+^ ions entered the A-site as well as the B-site, making a dual contribution to the ferroelectric and piezoelectric properties, having both softening and hardening effects in our KNN samples.

Finally, the results in Table 2 reveal that the acoustical impedance of the KNN ceramics is much smaller than that of the PZT system (~ 30 MRayl), which is very interesting from the point of view of piezoelectric ultrasonic transducers for biomedical images.

## 4. Conclusions

High density single-phase (ρ_th_ ~ 90%) (1−*x*)BaTiO_3_-(*x*)NaNbO_3_ (BTNN), 0.10 ≤ *x* ≤ 90, and “pure”, Li- (ρ_th_ ~ 97%) and Cu-doped (K_0.48_, Na_0.52_)NbO_3_ (ρ_th_ ~ 99.5%) lead free ceramics were prepared by different sintering processes. Dry high-energy ball milling (HEBM), following thermal treatment (calcining and sintering), was used for BTNN ceramics, while the KNN samples were densified by SPS sintering. Concentration dependent and pseudo tetragonal (*P*4*mm*) structured BTTN ceramics, with enhanced dielectric, ferroelectric and potentially exploited shear piezoelectric properties (*g_15_* ~ 65 × 10^−3^ Vm/N and *d_15_* ~ 630–930 × 10^−12^ m/V piezoelectric parameters) were obtained. It was verified that the addition of Li and Cu elements decreases, by around 100 °C, the sintering temperature of KNN ceramics. The ferroelectric (polarization) and piezoelectric (quality factor) properties of the KNN ceramics showed that Cu^2+^ was incorporated into the A and B sites of the perovskite structure, having both hardening and softening effects. Further improvements in the processing routes should be made to effectively control the site occupancy by Cu^2+^ ions, in order to obtain reproducible properties.

## Figures and Tables

**Table 1:** Temperature of the maximum of the dielectric constant (*T*_m_) and relative dielectric permittivity (ε), piezoelectric coefficients (*d*, *g*), acoustic impedance (*Z*) and quality factor (*Q*) measured at room temperature for (1−*x*)BaTiO_3_-(*x*)NaNbO_3_ ceramic samples and PZT: PZT-EC-64 (Type I).

Parameters	*x* = 0.70	*x* = 0.80	*x* = 0.90	PZT
T_m_ (K)	298	352	612	598
$\varepsilon_{33}^{T}$	2342	1240	1030	1300
*k*_31_	0.022	0.013	0.012	0.33
*k*_33_	0.016	0.015	0.013	0.70
*k*_15_	0.57	0.55	0.66	0.71
*k_t_*	0.02	0.014	0.013	0.51
*d*_31_ (10^−12^ m/V)	10.5	1.7	6.0	123
*d*_33_ (10^−12^ m/V)	5.0	3.1	3.3	289
*d*_15_ (10^−12^ m/V)	937	632	561	496
*g*_31_ (10^−3^ Vm/N)	0.49	0.15	0.66	11
*g*_33_ (10^−3^ Vm/N)	0.23	0.28	036	26
*g*_15_ (10^−3^ Vm/N)	47	65	64	39
*Q_m_*	119	182	217	500

**Table 2:** Ferroelectric-paraelectric transition temperature (*T*_C_) and relative dielectric permittivity (ε), piezoelectric coefficients (*d*, *g*), acoustic impedance (*Z*) and quality factor (*Q*) measured at room temperature for KNN (“pure”, KNN + Li and KNN + Cu) ceramic samples.

Parameters	KNN	KNN + Li	KNN + Cu
*T*_C_ (K)	406	454	420
$\varepsilon_{33}^{T}$	1654	400	420
*k_p_*	0.36	0.39	0.35
*k_t_*	0.39	0.30	0.28
*n_p_* (m/s)	3439	3165	3510
$n_{t}$ (m/s)	2988	2686	2799
*Q_pm_*	166	114	112
$Q_{tm}$	15	15	14
*Z*_p_ (MRayl)	15.2	14.0	15.5
*Z*_t_ (MRayl)	13.2	11.9	12.4
